# Dynamic human and avatar facial expressions elicit differential brain responses

**DOI:** 10.1093/scan/nsaa039

**Published:** 2020-03-30

**Authors:** Lorena C Kegel, Peter Brugger, Sascha Frühholz, Thomas Grunwald, Peter Hilfiker, Oona Kohnen, Miriam L Loertscher, Dieter Mersch, Anton Rey, Teresa Sollfrank, Bettina K Steiger, Joerg Sternagel, Michel Weber, Hennric Jokeit

**Affiliations:** 1 Swiss Epilepsy Center, CH-8008 Zurich, Switzerland; 2 Department of Psychology, University of Zurich, Zurich, Switzerland; 3 Neuropsychology Unit, Valens Rehabilitation Centre, Valens, Switzerland; 4 Institute for the Performing Arts and Film, Zurich University of the Arts, Zurich, Switzerland; 5 Institute for Critical Theory, Zurich University of the Arts, Zurich, Switzerland; 6 Department of Psychiatry, Psychotherapy, and Psychosomatics, University Hospital of Psychiatry Zurich, Zurich, Switzerland; 7 Department of Psychology, University of Bern, Bern, Switzerland

**Keywords:** face, emotion, avatar, computer-generated character, fMRI

## Abstract

Computer-generated characters, so-called avatars, are widely used in advertising, entertainment, human–computer interaction or as research tools to investigate human emotion perception. However, brain responses to avatar and human faces have scarcely been studied to date. As such, it remains unclear whether dynamic facial expressions of avatars evoke different brain responses than dynamic facial expressions of humans. In this study, we designed anthropomorphic avatars animated with motion tracking and tested whether the human brain processes fearful and neutral expressions in human and avatar faces differently. Our fMRI results showed that fearful human expressions evoked stronger responses than fearful avatar expressions in the ventral anterior and posterior cingulate gyrus, the anterior insula, the anterior and posterior superior temporal sulcus, and the inferior frontal gyrus. Fearful expressions in human and avatar faces evoked similar responses in the amygdala. We did not find different responses to neutral human and avatar expressions. Our results highlight differences, but also similarities in the processing of fearful human expressions and fearful avatar expressions even if they are designed to be highly anthropomorphic and animated with motion tracking. This has important consequences for research using dynamic avatars, especially when processes are investigated that involve cortical and subcortical regions.

## Introduction

While we are becoming more experienced with computer-generated characters, or avatars, that are used in animated films, social media or as human–computer interfaces, we do not know how we react and adapt to interactions with our virtual counterparts (Kätsyri *et al.,* 2017; Hsu, 2019). The use of avatars in entertainment and commercial settings is accompanied by an increasing use of avatars to investigate emotion perception since avatars enable highly standardized experiments that resemble real-life social situations (Zaki and Ochsner, 2009; Crookes *et al.,* 2015; de Borst and de Gelder, 2015). As a result, facial expressions of avatars have been shown to influence human decision making and cooperative behavior, which is crucial for the commercial use of avatars (Choi *et al*., 2012; de Melo *et al*., 2011a, 2011b; Scherer and Von Wangenheim, 2014). Hence, it seems vital to investigate the underpinnings of human behavior and associated brain processes during interactions with human-like avatars (Cross, Hortensius, *et al*., 2019; Epley *et al.,* 2008). In the present study, we investigate whether brain activation differs in response to dynamic human facial expressions and dynamic avatar facial expressions, and if so, which brain regions show activation differences.

Central to the processing of facial expressions and facial identity is a distributed network of brain regions that responds more strongly to human faces than to other visual information (Dricu and Frühholz, 2016; Fernández Dols and Russell, 2017). In this neural face perception network, the posterior and anterior superior temporal sulcus (STS) as well as the inferior frontal gyrus (IFG) form a dorsal pathway sensitive to dynamic features of faces like facial motion and gaze. Conversely, the inferior occipital gyrus, the fusiform gyrus (FG) and the anterior temporal lobe comprise the ventral pathway where invariant features of faces like form and configuration are processed (Haxby *et al.,* 2000; Duchaine and Yovel, 2015).

Based on the functional characterization of these brain regions for face perception, one may hypothesize that differences in brain responses to dynamic human and avatar expressions depend on the specific functions of these pathways. Thus, the ventral pathway, which is tuned to invariant facial features, may respond equally to anthropomorphic avatar faces and their human counterparts. Especially the FG may show equal responses to human and avatar faces, given its importance in the holistic processing of the facial form, independent of motions and emotions. On the other hand, the dorsal pathway may be activated differently by dynamic human and avatar facial expressions. Computer-generated faces often lack subtle dynamic features, such as expression-related wrinkles. Since dorsal regions are mainly engaged in the processing of facial motion, it is plausible that the STS and the IFG show stronger responses to dynamic human expressions compared to dynamic avatar expressions.

In addition to the cortical pathways discussed above, previous research has identified a subcortical route that is particularly involved in the processing of emotional facial expressions (Haxby *et al.,* 2000; Vuilleumier, 2005). This subcortical face processing route is formed by the amygdala, together with the pulvinar and the superior colliculus and may precede responses to dynamic human expressions in the ventral temporal cortex (Johnson, 2005; Méndez-Bértolo *et al.,* 2016). It has been proposed that this rapid subcortical processing is made possible by a magnocellular channel to the amygdala that is tuned to low-spatial frequency input. Typically, low-spatial frequency input provides information about coarse stimulus features like the configuration or form of a face. Conversely, a slower parvocellular channel to face-sensitive cortical regions is attuned to high-spatial frequency information in faces. This fine-grained parvocellular input thus provides slow but high-resolution information about local features of faces like expression-related wrinkles (Vuilleumier *et al.,* 2003; Kumar and Srinivasan, 2011; Dima *et al.,* 2018).

Although the exact functional role of the subcortical route in face perception remains controversial (Pessoa and Adolphs, 2010; McFadyen *et al.,* 2017), it is assumed that it enables the fast detection of fear- or threat-related environmental signals in the absence of slower cortical processing (LeDoux, 2000; Adolphs, 2001; Vuilleumier *et al.,* 2003). To date, no study has investigated whether the subcortical route that conveys low-spatial frequency information to the amygdala also mediates the processing of dynamic human and avatar expressions. It is thus not known whether amygdala responses to dynamic human and avatar expressions differ from cortical responses. In general, we would assume that human and avatar faces both entail coarse low-spatial frequency information about face configuration and form activating the amygdala. However, the composition and the range of the spatial frequency spectrum of avatar faces may depend on their level of elaboration (e.g. detectable wrinkles or not) and thus may differ from human faces with a broad spatial frequency spectrum.

Given the increasing use of avatars, several behavioral and imaging studies have investigated processing differences between human and avatar facial expressions. On a behavioral level, previous studies have shown that facial expressions are reliably recognized in both static and dynamic human and avatar faces (Dyck *et al.,* 2008; Gutiérrez-Maldonado *et al.,* 2014). On a neural basis, however, ventral and dorsal regions of the face perception network (Haxby *et al.,* 2000; Duchaine and Yovel, 2015) showed stronger responses to static human expressions than to static avatar expressions (Moser *et al.,* 2007; James *et al.,* 2015; Kätsyri *et al.,* 2020). More precisely, the FG, the STS and the IFG were more activated by static human than avatar expressions (Moser *et al.,* 2007; James *et al.,* 2015; Kätsyri *et al.,* 2020). Former results on amygdala responses to human and avatar facial expressions are mixed. Whereas two studies comparing static emotional expressions in human and avatar faces found no significant differences in amygdalar responses (Moser *et al.,* 2007; Kätsyri *et al.,* 2020), another study using neutral pictures of human and cartoon faces showed a stronger response of the amygdala to human faces (James *et al.,* 2015).

These results indicate that human and avatar facial expressions are not processed in the same way in both dorsal and ventral regions of the face perception network. Moreover, there is also a processing difference between cortical and subcortical regions that may be attributed to their differential sensitivity to certain ranges of spatial frequency. Yet, those results have been obtained using static facial expressions. So, virtually nothing is known about the differential processing of dynamic human and avatar facial expressions. To help closing this gap, we assessed brain responses to dynamic facial expressions of actors and their customized avatar look-alikes, which have been developed for this study. During the acquisition of functional MRI data, participants watched short videos of fearful and neutral expressions of the actors and avatars. By asking our participants to rate the intensity of the presented expressions within 2 weeks after the scanning session, we were able to investigate whether the intensity of the expressions also influences brain activation.

Based on previous results with static avatar expressions (Moser *et al.,* 2007; James *et al.,* 2015; Kätsyri *et al.,* 2020) and the role of the dorsal pathway for dynamic information in face perception (Haxby *et al.,* 2000; Duchaine and Yovel, 2015), we expected the STS and the IFG to show stronger responses to dynamic human facial expressions than to dynamic avatar facial expressions. Furthermore, we presumed that the processing difference between dynamic human and avatar faces should be larger for fearful expressions than for neutral expressions. We expected such an interaction effect to be present in the STS and the IFG because those regions are sensitive to dynamic features of facial expressions, which characterize fearful more than neutral expressions.

## Materials and methods

### Participants

We recruited 30 healthy participants aged between 18 and 62 years (16 female; *M*_age_ = 39.98 years; SD_age_ = 12.38 years) who reported no history of psychiatric or neurological disorders. During data collection, we had to exclude four participants from the final analyses for various reasons including excessive movement, vigilance problems and discomfort (see Supplementary material). This resulted in a final sample of 26 participants aged between 18 and 62 years (13 female; *M*_age_ = 40.64 years; SD_age_ = 12.24 years). Ethical approval for this study was obtained by the local ethics committee and subjects were only tested after given their written informed consent in line with the Declaration of Helsinki.

### Stimuli

**Fig. 1:**
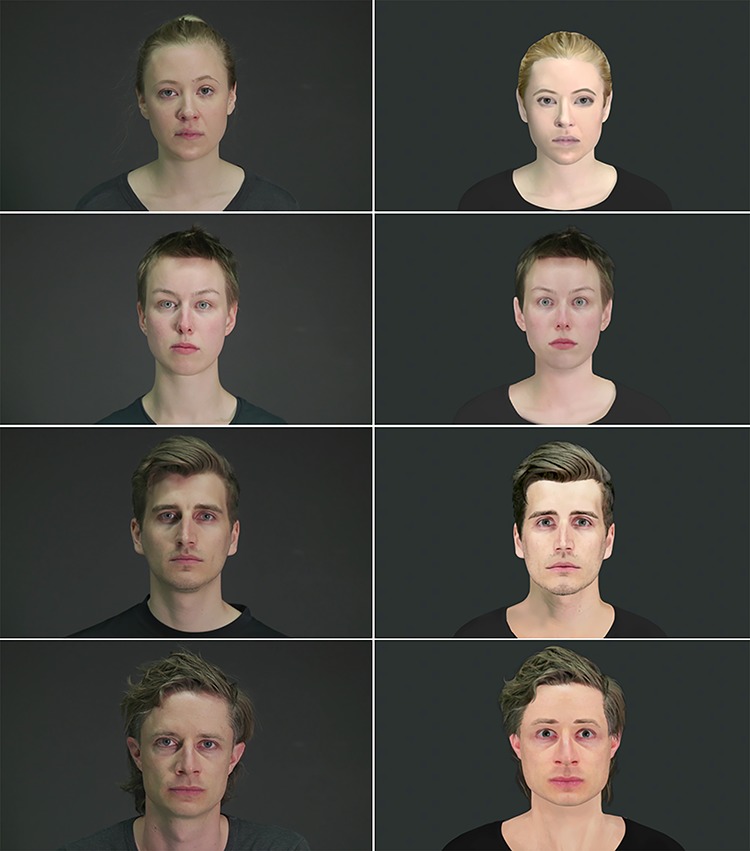
Depiction of the female actors on the upper left and the male actors on the lower left. Their respective avatars are shown on the right.

We used a set of videos that have been developed for the study in a three-step process in cooperation with the Zurich University of the Arts: (i) fearful and neutral human facial expressions were recorded from four actors, (ii) for each actor, a customized avatar was created by a graphic artist (Pinterac SARL, France) to match their appearance (see Figure 1), (iii) by motion tracking with 66 tracking points (FaceRig©, Holotech Studios SRL, Romania), the actors’ recorded expressions were conveyed onto the avatar faces (see Supplementary material). For each actor and each avatar, eight fearful and eight neutral videos were shown during the scanning session, resulting in a total of 128 videos, each lasting 3 s.

In addition, we constructed 128 scrambled versions of these videos by using a 40 × 45 grid and randomly scrambling the grid squares with MATLAB 2017a. Thus, we obtained a set of videos containing dynamic information equivalent to the videos with fearful expressions, but without emotional or facial attributes. This condition enabled us to test whether BOLD responses associated with dynamic fearful expressions can be ascribed to the mere processing of low-level dynamic visual information. The set of scrambled videos was divided into two subsets of 64 videos each and one of the two subsets was assigned pseudo-randomly (in the sequence of scanning) to each participant.

### Procedure

**Fig. 2:**
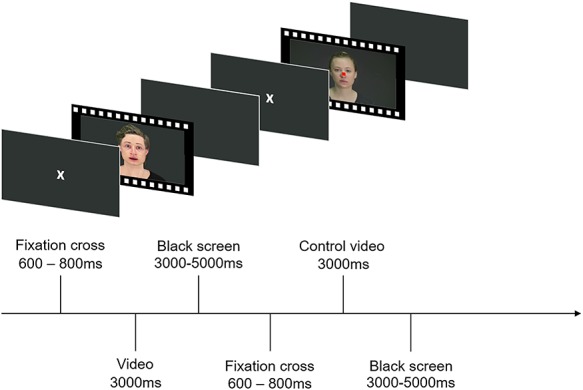
Exemplary depiction of the procedure during the two functional runs. After a fixation cross, a video showing either a human or an avatar facial expression, a scrambled video or a control video was displayed. Participants were instructed to passively watch the videos and to respond with a button press when a video with a red square appeared. After each video a black screen was shown.

Before scanning, participants were familiarized with the fMRI task. They were instructed that they would see videos of human and avatar faces showing fearful or neutral expressions and videos with scrambled patterns. They were told to watch the displayed faces or scrambled patterns. Occasionally, a video with a red square centered on the displayed face or scrambled pattern would appear to which they would have to respond with a button press. This task was used to ensure participants’ attention. The simplicity of the task, however, also ensured that the participants could naturally track the videos. Participants completed two functional runs while passively viewing 32 videos of fearful expressions (16 human, 16 avatar), 32 videos of neutral expressions (16 human, 16 avatar), 32 scrambled videos and 8 videos with a red square (control trials). This resulted in a total of 104 trials per run and 208 trials per session. The total number of button presses and the response times were recorded. Trials were randomly presented in an event-related design with jittered intertrial intervals (see Figure 2). After scanning, participants were reimbursed with 30 Swiss Francs and reminded that they were asked to rate the videos of human and avatar expressions within 2 weeks in an online rating survey according to their intensity. Each survey contained on average 16 videos showing fearful human expressions, 16 videos showing fearful avatar expressions and two videos showing neutral human expressions, as well as two videos showing neutral avatar expressions as a control condition (see Supplementary material). After each video, the intensity of the facial expression had to be rated on a scale from 1 (not very intense) to 6 (extremely intense).

### MRI acquisition and processing

#### Apparatus and acquisition parameters

Stimuli were presented using Cogent 2000 (version: 1.32) under MATLAB 2015a. Videos were displayed via a back-projection that was visible by a mirror on the head coil (visual angle of the faces: 8.5° vertical, 7° horizontal). MRI scans were obtained on a 3 Tesla Philips Achieva scanner (Philips Medical Systems, Best, The Netherlands) using a 32-channel head coil. We acquired structural images covering the whole brain using a T1-weighted MPRAGE sequence with the following parameters: TR = 8.1 ms, TE = 3.7 ms, slices = 176 sagittal slices, voxel size = 1 × 1 × 1 mm, matrix size = 240 × 164 mm, FOV = 240 × 240 mm, flip angle = 8°, no fat suppression, total acquisition time = 05:37. To track BOLD responses in the whole brain, we used an EPI sequence with 32 sequential ascending axial slices co-planar to the AC–PC line and an interslice gap of 0.4 mm (TR = 1800 ms, TE = 30 ms, voxel size = 2.75 × 2.75 × 3.5 mm, matrix size = 80 × 82 mm, FOV = 222 × 222 mm, flip angle = 75°, total acquisition time = 14:22). The first 10 volumes of each run were discarded by the scanner to allow the equilibration of T1 saturation effects so that in total 467 volumes per run were acquired.

#### MRI preprocessing

We analyzed functional data using SPM12 (version 6906; Wellcome Trust Centre for Neuroimaging, London, UK; http://www.fil.ion.ucl.ac.uk/spm/) on MATLAB 2017a. Functional images were realigned to the first image in the series, corrected for slice timing to the middle slice using sinc interpolation, and the mean functional image was co-registered to the individual anatomical image. Next, the anatomical scans were segmented into different tissue types and normalized to the Montreal Neurological Institute template using DARTEL (Ashburner, 2007). During this step, a mean anatomical template for the whole group was generated. Functional images were resampled to a voxel size of 2 × 2 × 2 mm and spatially smoothed (8 mm full-width half-maximum Gaussian kernel). After pre-processing, the realignment parameters were reviewed and participants moving >2 mm in either x-, y- or z-direction were excluded from further analyses.

#### Imaging analysis

Statistical analyses at the first level were performed with a general linear model across the whole brain. High-pass temporal filtering was set to a cut off of 128 s to filter out low-frequency noise. Individual trials were modeled with the SPM12 default canonical hemodynamic response function defined by the onset and duration of the videos. We modeled the following conditions as regressors of interest: condition face type (Human > Avatar), condition facial expression (Fear > Neutral), as well as condition scramble (non-scrambled > scrambled). Control trials were also modeled as regressors of interest but excluded for group-level analyses. To minimize false-positive findings related to task-correlated motion, realignment parameters were included as regressors of no interest.

To obtain results at group level, we analyzed first-level contrast images with one-sample *t*-tests. Median rating differences per participant were included as covariates to account for potential BOLD response differences due to dissimilar intensity levels of the videos (see section behavioral analysis). As one participant had not completed the rating survey, the median rating difference of the other participants was used as a substitute. Brain activation patterns bigger than a cluster extent of *k* = 5 and remaining under a voxel-wise FWE corrected *P*-value of <0.05 will be presented in the results section.

#### Defining regions of interest

In addition to the whole-brain analysis, a region-of-interest (ROI) analysis was performed in order to limit the analysis to groups of voxels that have been shown to be functionally coherent in previous studies. The following independent ROIs were a priori defined based on previous literature and using the Neuromorphometrics atlas implemented in SPM12 (http://www.neuromorphometrics.com/): the FG, the STS, the IFG and the amygdala. Based on the group-level statistical parametric maps, average ROI signals were extracted and compared for the different conditions using MarsBaR (Brett *et al.,* 2002). The resulting *t*-test outcomes were considered significant if they were below a Bonferroni-corrected statistical threshold of *P* < 0.05.

### Behavioral analysis

We examined the intensity ratings of human and avatar facial expressions with the exact Wilcoxon test using SPSS (Version 23). First, we analyzed median differences between ratings of fearful and neutral facial expressions separately for human and avatar videos. Second, we investigated median differences between ratings of fearful human and fearful avatar facial expressions as well as median differences between ratings of neutral human and neutral avatar facial expressions. To test for rating differences associated with the gender of the displayed character, we computed median differences between videos with male and female characters separately for human and avatar videos (see Supplementary material).

## Results

### Behavioral responses

To control subject’s task engagement during the scanning session, participants were required to respond with a button press to infrequent presented control videos with a red square centered on the displayed face or scrambled pattern. Average detection rate of control videos was nearly perfect (99%) with a median response time of 754 ± 246 ms.

The statistical analysis of the rating of fearful and neutral videos showed that fearful human expressions were rated as more intense (Mdn = 5) than neutral human expressions (Mdn = 2; exact Wilcoxon test: *z* = −4.47, *P* < 0.001). The same difference was observable for avatar faces, with fearful avatar expressions rated as more intense (Mdn = 3) in comparison to neutral avatar expressions (Mdn = 2; exact Wilcoxon test: *z* = −3.16, *P* < 0.001). Fearful human expressions were judged as more intense (Mdn = 5) than fearful avatar expressions (Mdn = 3; exact Wilcoxon test: *z* = −4.31, *P* < 0.001). No significant difference was apparent for neutral human and neutral avatar expressions (exact Wilcoxon test: *z* = −1.19, *P* = 0.244). Analysis results of the rating of female versus male faces are shown in Supplementary Table S1.

### BOLD response to human and avatar facial expressions

**Fig. 3:**
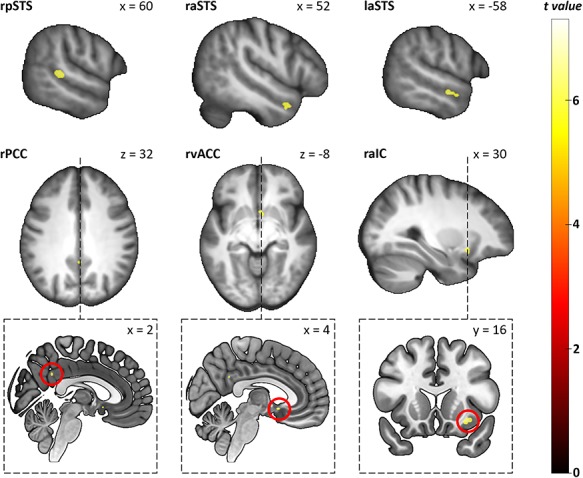
Group-level statistical parametric maps showing stronger responses to fearful human than to fearful avatar facial expressions (voxel-wise *P*-FWE < 0.05). For better illustration, the clusters are shown on the mean anatomical template of the study population (top and middle row) and a part of them also on the ‘mni152_2009bet’ template from MRIcroGL (bottom row). Small clusters (*k* < 15) are highlighted with dashed lines (middle row) indicating the corresponding sagittal or coronal section (bottom line), where the clusters are highlighted with red circles. r, right; l, left; pSTS, posterior superior temporal sulcus; aSTS, anterior superior temporal sulcus; PCC, posterior cingulate cortex; vACC, ventral anterior cingulate cortex; aIC, anterior insular cortex.

**Fig. 4:**
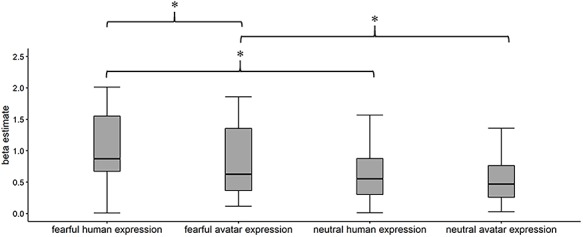
Distribution of beta estimates per condition showing both main effects (face type and face expression) and the interaction effect in the right STS. Whiskers indicate the 25th and the 75th percentile. ^*^P < 0.05 Bonferroni corrected.

**Fig. 5:**
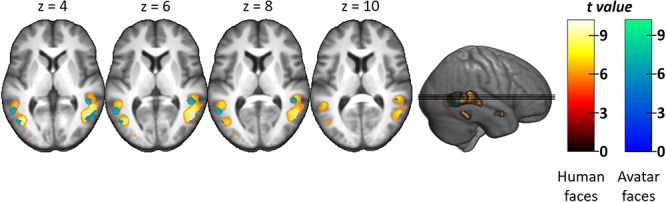
Group-level statistical parametric maps showing the response difference between human and avatar faces for fearful expressions versus neutral expressions in bilateral posterior STS (voxel-wise *P*-FWE < 0.05). Note that the direct comparison of the clusters was significant only for the right posterior STS in the ROI analysis and not in the whole-brain analysis. Therefore, the whole-brain clusters shown serve descriptive purposes only.

First, we contrasted fearful human and avatar facial expressions across the whole brain. Fearful human expressions elicited stronger responses in the posterior and anterior STS, the anterior insular cortex (aIC), as well as in the posterior (PCC) and ventral anterior cingulate cortex (vACC; see Table 1 and Figure 3). The analysis of the a priori defined independent ROIs revealed that fearful human expressions elicited stronger responses than fearful avatar expressions in bilateral STS (left: *T* = 4.75, *p*_corr_ < 0.001, right: *T* = 5.36, *p*_corr_ < 0.001) and bilateral IFG (left: *T* = 3.14, *p*_corr_ = 0.022, right: *T* = 2.81, *p*_corr_ = 0.047). Second, we contrasted neutral human and avatar expressions with one another. They did not evoke significantly different responses in either the whole-brain analysis or the ROI analysis. The interaction contrast face type (human versus avatar) × facial expression (fearful versus neutral) revealed that the right STS showed a larger response difference between human and avatar faces for fearful expressions than for neutral expressions (ROI analysis: *T* = 2.8, *p*_corr_ = 0.050, see Figure 4 for distribution of beta estimates per condition and Figure 5 for a direct comparison of the whole-brain activation cluster in bilateral STS).

**Table 1:** Clusters showing a significant difference in BOLD response to fearful human expressions compared to fearful avatar expressions in the second level whole-brain analysis

Brain area	Side	*k*	MNI coordinates			*T*-value	*P*-FWE
			*x*	*y*	*z*		
STS, posterior	R	50	60	−36	6	6.69	0.009
			64	−46	8	6.29	0.020
STS, anterior	R	23	56	2	−20	7.27	0.003
			52	8	−24	6.49	0.013
STS, anterior	L	21	−58	−10	−12	6.87	0.006
			−58	−2	−14	5.93	0.040
Cingulate gyrus, posterior	R	5	2	−48	32	6.02	0.033
Cingulate gyrus, ventral anterior	R	10	4	10	−8	6.42	0.015
Insular cortex, anterior	R	14	30	16	−12	6.90	0.006

*Notes.* Voxel-wise FWE corrected *p*-value is shown. Missing values under *k* indicate that the activation peak of the respective brain area pertains to the above-mentioned cluster. In cases where several activation peaks belonged to one cluster, the brain areas were listed according to the size of their *T*-value. STS = superior temporal sulcus.

### BOLD response to fearful and neutral facial expressions

**Fig. 6:**
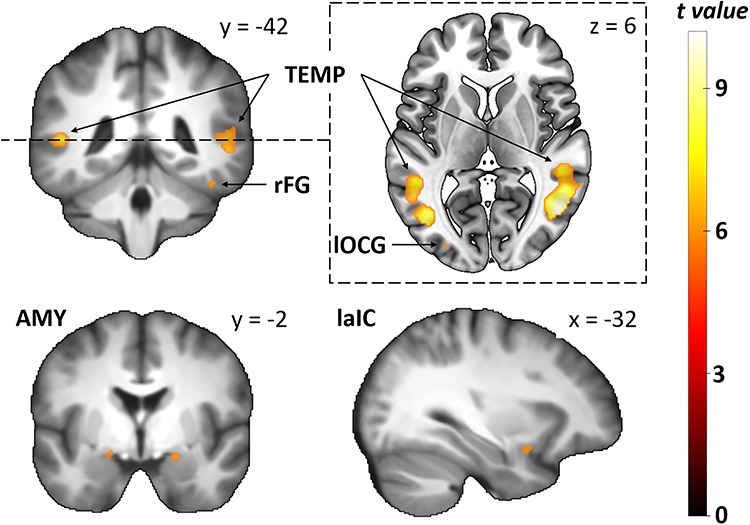
Group-level statistical parametric maps showing stronger responses to fearful human than to neutral human expressions (voxel-wise *P*-FEW < 0.05). For better illustration, the clusters are shown on the mean anatomical template of the study population (top left and bottom row) and on the ‘mni152_2009bet’ template from MRIcroGL (top right). Images in the top row show coronal and axial sections of the clusters in bilateral inferior occipital cortex (see TEMP and lOCG) as well as in ventral and dorsal temporal cortex (see TEMP and rFG). Images in the bottom row show clusters in bilateral amygdala and left anterior insular cortex. TEMP = bilateral dorsal temporal cortex; rFG = right fusiform gyrus; lOCG = left inferior occipital gyrus; AMY = bilateral amygdala; laic = left anterior insular cortex.

**Fig. 7:**
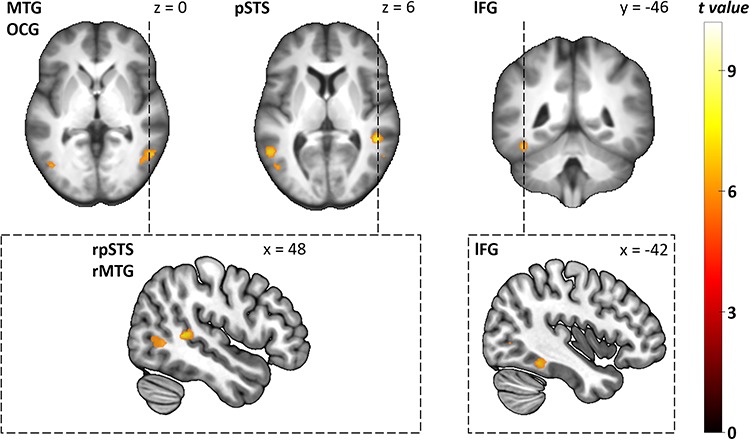
Group-level statistical parametric maps showing stronger responses to fearful avatar than to neutral avatar expressions (voxel-wise *P*-FEW < 0.05). For better illustration, the clusters are shown on the mean anatomical template of the study population (top row) and on the ‘mni152_2009bet’ template from MRIcroGL (bottom row). Images on the top left, top middle and bottom left show axial and sagittal sections of the clusters in bilateral inferior occipital gyrus and dorsal temporal cortex. Images on the top right and bottom right show coronal and sagittal sections of the cluster in the ventral temporal cortex. r, right; l, left; MTG, middle temporal gyurs; OCG, inferior occipital gyrus; pSTS, posterior superior sulcus; FG, fusiform gyrus.

Next, we contrasted fearful and neutral expressions displayed in human faces across the whole brain. Fearful human expressions evoked stronger responses in bilateral inferior occipital cortex, ventral and dorsal temporal cortex, the left aIC and bilateral amygdalae (see Table 2 and Figure 6). The ROI analysis revealed that fearful human expressions evoked stronger responses than neutral human expressions in bilateral amygdala (left: *T* = 4.12, *p*_corr_ = 0.002, right: *T* = 5, *p*_corr_ < 0.001), right occipital FG (*T* = 3.2, *p*_corr_ = 0.019), bilateral STS (left: *T* = 4.97, *p*_corr_ < 0.001, right: *T* = 6.32, *p*_corr_ < 0.001) and bilateral IFG (left: *T* = 3.62, *p*_corr_ = 0.007, right: *T* = 3.58, *p*_corr_ < 0.007). We then contrasted fearful and neutral avatar expression across the whole brain. Fearful avatar expressions elicited stronger responses in bilateral inferior occipital cortex as well as ventral and dorsal temporal cortex (see Table 3 and Figure 7). The ROI analysis showed that fearful avatar expressions elicited stronger responses than neutral avatar expressions in the left amygdala (*T* = 3.27, *p*_corr_ = 0.016) and right STS (*T* = 3.35, *p*_corr_ = 0.013).

**Table 2:** Clusters showing a significant difference in BOLD response to fearful human expressions compared to neutral human expressions in the second level whole-brain analysis

Brain area	Side	*k*	MNI coordinates			*T*-value	*P-*FWE
			*x*	*y*	*z*		
Inferior occipital gyrus	R	816	48	−64	6	10.06	<0.001
Middle temporal gyrus, posterior	R		54	−50	4	9.48	<0.001
STS, posterior	R		52	−36	8	7.77	<0.001
FG, middle	R	24	46	−42	−20	6.35	0.015
Inferior occipital gyrus	L	5	−30	−88	6	6.39	0.014
Inferior occipital gyrus	L	413	−42	−68	2	10.09	<0.001
STS, posterior	L		−48	−46	8	8.07	<0.001
Insular cortex, anterior	L	6	−32	6	−16	5.96	0.033
Amygdala	R	27	24	2	−16	6.18	0.021
Amygdala	L	6	−18	−2	−12	6.03	0.028

*Notes.* Voxel-wise FWE corrected *p*-value is shown. Missing values under *k* indicate that the activation peak of the respective brain area pertains to the above-mentioned cluster. In cases where several activation peaks belonged to one cluster, the brain areas were listed according to the size of their *T*-value. STS = superior temporal sulcus; FG = fusiform gyrus.

**Table 3:** Clusters showing a significant difference in BOLD response to fearful avatar expressions compared to neutral avatar expressions in the second level whole-brain analysis

Brain area	Side	*k*	MNI coordinates			*T*-value	*P*-FWE
			*x*	*y*	*z*		
Middle temporal gyrus, posterior	R	105	56	−60	0	6.98	0.004
Inferior occipital gyrus	R		46	−64	2	6.15	0.023
STS, posterior	R	53	48	−42	6	7.65	0.001
Inferior occipital gyrus	L	35	−48	−70	4	6.70	0.007
STS, posterior	L	59	−56	−54	6	7.33	0.002
FG, middle	L	38	−42	−46	−18	6.89	0.005

*Notes.* Voxel-wise FWE corrected *P*-value is shown. Missing values under *k* indicate that the activation peak of the respective brain area pertains to the above-mentioned cluster. In cases where several activation peaks belonged to one cluster, the brain areas were listed according to the size of their *T*-value.

In favor of a concise presentation of our main results, whole-brain analyses concerning the scrambled videos are summarized in Supplementary Tables S2–S5. In short, contrasts between non-scrambled videos and their scrambled versions generally showed stronger responses in the inferior occipital cortex as well as in the ventral and dorsal temporal cortex to non-scrambled videos. Non-scrambled videos of human and avatar fearful expressions also evoked stronger responses in the amygdala than their scrambled versions.

## Discussion

### Summary

We set out to investigate whether dynamic facial expressions displayed by avatar faces are processed within the same cortical and subcortical brain regions known to be involved in the processing of dynamic human facial expressions. To this aim, we assessed the brain topography of BOLD responses to dynamic human and avatar faces displaying either fearful or neutral expressions. Using videos of actors and their customized avatars derived from the same videos by motion tracking, we were able to demonstrate that the topography and the magnitude of brain response differed between human and avatar faces, yet only when fearful expressions were presented. Our imaging analysis revealed predominantly right-sided brain areas that showed stronger responses to fearful human expressions compared to fearful avatar expressions. This cluster entailed cortical areas such as bilateral posterior and anterior parts of the superior temporal region, the IFG, the right PCC and vACC, as well as the right aIC. For the amygdala, a subcortical core region in face and emotion perception, no response difference was found when comparing fearful expressions between human and avatar faces. Furthermore, our analysis revealed an interaction effect in the right superior temporal region. That is, the response difference between human and avatar faces was larger for fearful than for neutral expressions in the right superior temporal region.

When comparing fearful and neutral expressions displayed by human faces, we were able to show stronger BOLD responses to fearful human expressions in dorsal and ventral regions of the face perception network as well as in the left aIC and bilateral amygdala. A qualitatively similar response pattern, but with fewer and smaller activation peaks, was found when comparing fearful and neutral expressions displayed by avatar faces. This cluster entailed posterior dorsal temporal regions, inferior occipital and fusiform regions, as well as the left amygdala.

### Modulation of brain activity by facial motion

In line with our hypothesis, we found stronger BOLD responses to fearful human expressions than to fearful avatar expressions in posterior and anterior parts of the superior temporal region and the IFG. These differential responses also appeared in comparison to scrambled videos, which indicates that the processing difference between fearful human and avatar expressions is not only associated with dynamic low-level visual features. This is in line with contemporary models of face perception, in which the superior temporal region and the IFG belong to the dorsal face processing pathway specialized for the processing of dynamic features of faces (Haxby *et al.,* 2000; Duchaine and Yovel, 2015). Since subtle motions like that of wrinkles were not detectable in our avatar faces for technical reasons, fearful avatar expressions may have evoked weaker BOLD responses in these regions compared to human expressions. However, the weaker BOLD responses to avatar expressions were also present when we modeled the intensity of facial expressions as covariate. Hence, it is more plausible that even when using motion tracking, avatar facial expressions resemble artificial facial motion that does not activate these regions in the same way as biological facial motion (Sarkheil *et al.,* 2013). Support for this hypothesis comes from a study investigating BOLD responses to biological motion in natural movie scenes and animated versions of these scenes. This study showed that areas implicated in the processing of biological motion such as the STS exhibit stronger responses to the same biological motion in natural movie scenes than in animated scenes (Mar *et al.,* 2007).

Furthermore, we observed at a descriptive level that the stronger responses to fearful human in contrast to fearful avatar faces were more apparent in the right brain hemisphere. This is in line with a recognition advantage for facial expressions containing high-spatial frequency information presented to the left visual field and thus processed in the right brain hemisphere (as opposed to the presentation in the right visual field and processing in the left brain hemisphere; Kumar and Srinivasan, 2011). Hence, the primarily right-sided responses we found may be associated with differences in the high-spatial frequency range between fearful human and avatar expressions.

In contrast to previous studies applying static avatar stimuli (Moser *et al.,* 2007; Kätsyri *et al.,* 2020), we found a similar BOLD response in the FG to fearful human and avatar expressions. Face perception models propose the FG to represent invariant face information such as form or identity (Haxby *et al.,* 2000; Duchaine and Yovel, 2015). Invariant facial information was present in both the human and avatar faces, which may be associated with the comparable activation pattern. Further support comes from studies showing that the FG was sensitive only to faces differing in typicality relative to an average face shape and that already a coarse representation of a face is sufficient to correctly identify it as a generic face (Said *et al.,* 2010; Goffaux *et al.,* 2011). Because our avatars were customized to the human face of their respective actor as closely as possible, such typicality is present in both the human faces and the avatar faces and may thus have equally activated the FG.

### Modulation of brain activity by emotion

To interpret our results, it is necessary to emphasize that we only found different BOLD responses to human and avatar faces when comparing fearful but not neutral expressions. Thus, emotional expressions exert an important influence on the processing of human and avatar faces. They activate the amygdala via a fast magnocellular channel conveying low-spatial frequency information and thus enabling rapid evaluation of general stimulus features (Vuilleumier *et al.,* 2003; de Gelder *et al.,* 2005; Vuilleumier and Pourtois, 2007; Adolphs, 2010). In line with this crude appraisal function of the amygdala, only coarse, typically blurred information is processed over this subcortical route (Vuilleumier, 2005). The presence of such coarse low-spatial frequency information in human and avatar faces may explain the comparable amygdala responses.

In addition, it is plausible that the amygdala has a modulatory effect on subsequent face perception via backward connections to cortical areas, such as the IFG and the STS (Furl *et al.,* 2013; Schirmer and Adolphs, 2017). Correspondingly, evidence was found for amygdala feedback loops to ventral and dorsal temporal areas during the processing of emotional facial expressions. These results indicate a modulatory effect of the amygdala on visual attention and successive processes underlying human face perception (Vuilleumier *et al.,* 2001; Furl *et al.,* 2013). Thus, our finding regarding stronger responses to fearful human than to fearful avatar expressions in dorsal temporal regions and the IFG may indicate complex visual coding of motion or gaze information present in fearful expressions and enhanced via feedback connections from the amygdala.

In contrast to our results, James *et al.* (2015) compared brain responses to neutral human and neutral cartoon faces and found stronger responses to human faces in the amygdala, the FG, the STS and the IFG. Possible explanations for these divergent findings are the use of static faces and bold differences between human faces and artificial cartoon faces. Accordingly, it has been shown that different levels of face realism elicit different neural responses as measured by EEG. Thus, the artificial appearance of computer-generated characters may change their processing (Schindler *et al.,* 2017). However, the avatars in the present study were more realistic and tailored to the individual actors’ faces.

### Are we empathic toward avatars?

Perceiving emotions in others almost inevitably elicites empathy in us, i.e. the attempt to understand and share the emotional state of another (Preston and de Waal, 2002). It is not yet known whether this can also be achieved by the perception of avatars. Although we did not assess empathy directly in our study, it may be possible to address this question cautiously based on the observed brain responses. We found stronger BOLD responses to fearful human expressions than to avatar expressions in the aIC, the vACC and the PCC. While the insula represents an integration hub for interoceptive processes and thus plays an important role in the perception of physiological signals within one’s own body (Craig, 2002; Garfinkel and Critchley, 2013), the PCC is linked (among other functions) with body ownership (Guterstam *et al.,* 2015). The vACC is further associated with implicit emotion regulation (Etkin *et al.,* 2015). Thus, different responses to fearful human and avatar facial expressions in the aforementioned brain regions may be explained by their association with empathy processes (Decety and Lamm, 2006; Bernhardt and Singer, 2012).

It is assumed that mirror neurons, which typically discharge when performing a certain motoric action as well as when the same motoric action is observed, are a potential basis for empathy-related brain responses (Gallese *et al.,* 1996; Rizzolatti and Sinigaglia, 2016). This so-called mirroring is also transferable to brain responses associated with experiencing an emotional state as well as observing the same emotional state in another individual (Rizzolatti and Sinigaglia, 2016). It may be hypothesized that observing fear in avatars evokes less mirroring in associated brain regions since they are easily identified as artificial or non-human and therefore less important for human interaction (Gu and Han, 2007; Fan and Han, 2008; Bernhardt and Singer, 2012; Rizzolatti and Sinigaglia, 2016). Empirical studies on empathy-related brain responses to computer-generated characters, however, are still rare. Most results stem from studies using robots and show that people empathize with robots and exhibit corresponding neural responses, albeit to a lesser extent compared to responses to humans.

Equivocally, we should consider that not empathy but rather processes related to emotional intelligence may account for the observed variance in brain responses and rating differences between human and avatar faces. It is possible that our lack of expertise with computer-generated faces is associated with lower measures of emotional intelligence regarding facial expressions of avatars. In line with this, previous studies have shown that measures of emotional intelligence are positively associated with anterior insula activity during face processing (Quarto *et al.,* 2016) and gray matter volume of the insula (Killgore *et al.,* 2012; Karle *et al.,* 2018). This must be considered when using avatars for research concerning social interactions (Bombari *et al.,* 2015; Cross, Riddoch, et al., 2019; Hortensius *et al.,* 2018; Rosenthal-von der Pütten *et al.,* 2014; Suzuki *et al.,* 2015).

### Limitations and future directions

Although our study contributes new findings, it has necessary limitations. First, we only used fearful and neutral facial expressions. Consequently, our results cannot be generalized to other facial expressions, as valence-dependent effects are possible (Dyck *et al.,* 2008; Fusar-Poli *et al.,* 2009). Second, differences to other studies may in part be due to the software applied to create and animate avatars. The quality of motion tracking of the employed software (FaceRig©) may be limited compared to future advances in software development. Another limitation is that the intensity rating was performed within 2 weeks after the scanning session. This may have affected the participants’ rating and thus our results are not comparable to studies in which participants provide their rating during the scanning session.

Future studies may clarify whether the complexity of computer-generated faces influences brain responses and recognition profiles of different emotional expressions. It is plausible that the absence of high-spatial frequency information in computer-generated faces represents a missing cue for emotion recognition. On the other hand, this absence could be advantageous because the facial expression may be less ambiguous. By using computer-generated faces showing only major features of facial expressions, we are able to generate tasks for emotion recognition with a low level of complexity (Dyck *et al.,* 2008). Such tasks may be especially beneficial for the training of emotion recognition in clinical populations considering the degree of their impairment (Dyck *et al.,* 2010; Arsalidou *et al.,* 2011; Szemere and Jokeit, 2015; Steiger and Jokeit, 2017). With increasing performance, the task may also be adjusted in difficulty by directly manipulating the perceptual features of the facial expressions (Macedo *et al.,* 2015). This possibility only applies to computer-generated characters and thus represents an important alternative to picture-based training.

## Conclusion

There are both differences and similarities in the patterns of brain responses to humans and avatars depending on the expressions shown. While amygdalar and cortical responses in ventral temporal regions do not differ in response to fearful human and avatar expressions, dorsal temporal and inferior frontal regions sensitive to dynamic facial information exhibit distinct responses. Together, this means that although avatar expressions evoke amygdala responses associated with the fast processing of emotions similar to human expressions, the artificial facial motion of avatars is likely not to be processed in the same way as human facial motion. Furthermore, empathy-related processes or processes associated with emotional intelligence may interplay with this. These interpretations may have important consequences for the implementation of avatars in various commercial and public applications and research. Nevertheless, we do not believe that the current findings imply that computer-generated faces are of no use considering their methodological benefits. Instead, we consider it necessary that researchers acknowledge these potential limitations and implement avatars according to the overall goal of the study.

## Supplementary Material

scan-19-409-File009_nsaa039
