# Supplementary material for: Dynamic human and avatar facial expressions elicit differential brain responses
Source: Soc Cogn Affect Neurosci. 2020 Mar 30;15(3):303–17. doi: 10.1093/scan/nsaa039 (PMC7235958; doi:10.1093/scan/nsaa039)
Supplement: scan-19-409-File009_nsaa039 [file scan-19-409-file009_nsaa039.docx]

Supplementary Material

Participants

Participants were recruited via three different sources: a volunteer database of the University of Zurich, a public advertisement in local social media and an internal advertisement targeted at the staff of the Swiss Epilepsy Center. Participants had to be able to follow and understand the information and the procedure of the study (i.e. no hindering language barrier, severe cognitive deficit, or psychiatric disease) and their vision was required to be normal or corrected to normal. To ensure MRI safety, participants had to fulfil standard MRI safety criteria (no pregnancy, no claustrophobia, no metallic implant or other metallic objects in the body that are contraindicated for MRI) and to remove all metallic or magnetic objects as well as electronic devices before the scanning session. Because of excessive and sudden movement during the scanning session (> 2mm), two female participants were excluded from the final analyses. Furthermore, one female participant was excluded from the final analyses because she exhibited marked vigilance problems. This was evident in the control condition, where she missed about one-third of the control trials. One male participant was excluded as he was unable to complete the scanning session because of marked discomfort due to the scanner noise.

Production of Face Stimuli

To create the stimulus set for this study, videos of neutral and fearful facial expressions were recorded with four Northern European actors aged between 26 and 31 years (two female; *M*_age_ = 28 years; *SD*_age_ = 2.38 years). All actors were or had been students of the Zurich University of the Arts and had medium to high acting experience. Both male actors and one of the actresses were recorded using a Canon C100 Mark II. The other actress was recorded using a Canon 5D Mark II. We controlled the color temperature of the light during the recordings with a panel light and held it constant at 3800 Kelvin.

All recordings were directed by the Head of the Institute for the Performing Arts and Film at the Zurich University of the Arts. Before recording fearful facial expressions, actors were instructed to imagine a situation in which they feel fearful and to immerse themselves in this situation and in the emotion felt. Further, they were instructed to express the emotion felt as intense but also as natural as possible. We refrained from instructing the actors to perform prototypical facial movements (e.g. with the use of action units (Ekman & Friesen, 1978)), in order to achieve a broad range of fearful facial expressions that appear as realistic as possible. Fearful facial expressions started off with neutral facial expressions where the actors looked motionless towards the camera. The neutral expressions were also recorded and used for the study. After the recordings, we transferred the facial expressions of each actor by motion tracking onto their custom-designed avatar using FaceRig^©^ (Holotech Studios SRL, Romania). For this purpose, the videos had to be loaded into FaceRig^©^ and its motion tracking system had to be calibrated to the input source, i.e. the corresponding face. The facial expressions were then tracked and the resulting avatar facial expressions were saved as output videos.

Selection of Face Stimuli

The first author of this manuscript initially reviewed all recordings and identified sections showing neutral or fearful facial expressions lasting 3 s. For this selection, the first author paid particular attention to the following criteria: i) actors show a fearful or neutral facial expression as intense as possible and hold the intensity of this expression for 3 s, ii) actors maintain a direct gaze towards the camera, iii) actors move their head or upper body only slightly so that eyes and mouth are always clearly visible, and iiii) actors do not cry or scream. Furthermore, two additional criteria were applied for the selection of neutral facial expressions: actors hold their mouth closed and do not blink. The selected sections were then cut to 3 s videos by a specialist in charge of video at the Zurich University of the Arts.

Subsequently, from the resulting 251 videos with neutral facial expressions and 217 videos with fearful facial expressions, the best 64 videos for the neutral condition (8 per actor and avatar) and the best 64 videos for the fearful condition (8 per actor and avatar) were selected. This was done by a second review of all videos by the first author. As part of this review, videos with fearful facial expressions were rated according to the intensity of the expression on a scale from 1 (*not very intense*) to 6 (*extremely intense*). Videos showing fearful facial expressions with a rating higher than 3 and fulfilling the criteria mentioned in the section above were chosen for the final stimuli set. No intensity rating was conducted for videos showing neutral facial expressions and only the above-mentioned criteria had to be met to include them in the final stimulus set.

Online Rating Survey and Behavioral Analysis

The online rating survey was programmed and carried out with the help of an online survey tool ([https://www.soscisurvey.de](https://www.soscisurvey.de/)). We constructed two parallel versions of the rating survey, each containing 64 videos showing fearful human and avatar facial expressions in random order. To shorten the time needed to complete the survey, we divided both parallel versions, resulting in four surveys with 32 different videos of fearful human and avatar facial expressions. Each survey also contained 4 control videos, which showed two neutral human and two neutral avatar facial expressions. These videos had been randomly selected from the stimulus set, but care was taken to always show two female and two male characters per survey. This resulted in a total of 16 different control videos (2 per actor and 2 per avatar). Participants had as much time as they needed to fill in each rating scale.

For the behavioral analysis, the rating distribution of each video was first visually inspected using boxplots. This visual inspection showed that about one quarter of the ratings for videos with fearful human and avatar facial expressions showed outliers. For this reason, for every participant we calculated the median intensity rating per condition (i.e. neutral human, fearful human, neutral avatar, and fearful avatar) instead of a mean intensity rating. The visual inspection of the data also showed that one participant had rated each video as *not very intense* (that is, either 1 or 2 out of six possible intensity levels selected). Since such a uniform rating performance seems implausible and may indicate unreliable completion of the rating survey, the median rating values of this participant per condition were substituted by the median rating values of the other participants. We conducted subsequent analyses to investigate median rating differences non-parametrically with the exact Wilcoxon test for two reasons: i) due to the remaining outliers in the ratings which were considered to be representative data points, ii) due to the unequal number of videos with fearful facial expressions compared to videos with neutral facial expressions.

| Supplementary Tables | | | | | | | | | | | | |
| --- | --- | --- | --- | --- | --- | --- | --- | --- | --- | --- | --- | --- |
| Supplementary Table S1 | | | | | | | | | | | | |
| *Results of Wilcoxon-Test comparing the ratings of intensity in female versus male fearful human and avatar expressions* | | | | | | | | | | | | |
|  |  | Negative ranks | | |  | Positive ranks | | |  | Test statistics | | |
| Video type |  | *n* | Mean rank | Sum of ranks |  | *n* | Mean rank | Sum of ranks |  | Ties | *z* | *p* |
| Fearful human faces |  |  |  |  |  |  |  |  |  |  |  |  |
| Female - male | | 9 | 7.78 | 70 |  | 5 | 7.0 | 35 |  | 12.0 | -1.16 | .290 |
| Fearful avatar faces |  |  |  |  |  |  |  |  |  |  |  |  |
| Female - male | | 15 | 10.27 | 154 |  | 5 | 11.2 | 56 |  | 6.0 | -1.86 | .064 |
| *Note.* Exact two-tailed significance is reported. | | | | | | | | | | | | |

As shown in Supplementary Table S1, female fearful human expressions (*Mdn* = 5) and male fearful human expressions (*Mdn* = 5) did not differ in terms of rated intensity. Likewise, no significant intensity difference was found between female fearful avatar expressions (*Mdn* = 2) and male fearful avatar expressions (*Mdn* = 3).

| Supplementary Table S2 | | | | | | | |
| --- | --- | --- | --- | --- | --- | --- | --- |
| *Clusters showing a significant difference in BOLD response to fearful human expressions compared to scrambled fearful human expressions in the second level whole-brain analysis* | | | | | | | |
| Brain area | Side | *k* | MNI coordinates | | | *T*-value | *p-FWE* |
|  |  |  | *x* | *y* | *z* |  |  |
|  |  |  |  |  |  |  |  |
| Inferior occipital gyrus | R | 1342 | 40 | -74 | -8 | 11.24 | < .001 |
| Fusiform gyrus | R |  | 44 | -50 | -20 | 11.15 | < .001 |
| Inferior occipital gyrus | R |  | 44 | -68 | -4 | 9.49 | < .001 |
| Temporal pole | R | 18 | 54 | 8 | -18 | 8.2 | < .001 |
| Inferior occipital gyrus | L | 6 | -42 | -74 | -2 | 6.58 | .009 |
| Fusiform gyrus | L | 158 | -42 | -48 | -18 | 9.29 | < .001 |
| Middle temporal gyrus,  posterior | L | 21 | -44 | -64 | 8 | 6.25 | .019 |
| Superior temporal sulcus, posterior | L | 120 | -48 | -46 | 8 | 8.11 | < .001 |
| Temporal pole | L | 88 | -34 | 4 | -20 | 7.78 | < .001 |
| Frontal operculum | L | 184 | -44 | 24 | -2 | 8.94 | < .001 |
| Amygdala | R | 98 | 22 | -2 | -12 | 7.86 | < .001 |
| Pallidum | L | 28 | -16 | -6 | -8 | 6.83 | .005 |
| *Note.* Voxel-wise FWE corrected *p*-value is shown. Missing values under *k* indicate that the activation peak of the respective brain area pertains to the above-mentioned cluster. In cases where several activation peaks belonged to one cluster, the brain areas were listed according to the size of their *T*-value. | | | | | | | |

| Supplementary Table S3 | | | | | | | |
| --- | --- | --- | --- | --- | --- | --- | --- |
| *Clusters showing a significant difference in BOLD response to neutral human expressions compared to scrambled neutral human expressions in the second level whole-brain analysis* | | | | | | | |
| Brain area | Side | *k* | MNI coordinates | | | *T*-value | *p-FWE* |
|  |  |  | *x* | *y* | *z* |  |  |
|  |  |  |  |  |  |  |  |
| Fusiform gyrus | R | 487 | 42 | -44 | -18 | 9.09 | < .001 |
| Inferior occipital gyrus | R |  | 46 | -66 | -2 | 8.23 | < .001 |
| Inferior occipital gyrus | R |  | 40 | -76 | -6 | 7.69 | < .001 |
| Fusiform gyrus | L | 110 | -42 | -48 | -20 | 8.6 | .002 |
| *Note.* Voxel-wise FWE corrected *p*-value is shown. Missing values under *k* indicate that the activation peak of the respective brain area pertains to the above-mentioned cluster. In cases where several activation peaks belonged to one cluster, the brain areas were listed according to the size of their *T*-value. | | | | | | | |

| Supplementary Table S4 | | | | | | | |
| --- | --- | --- | --- | --- | --- | --- | --- |
| *Clusters showing a significant difference in BOLD response to fearful avatar expressions compared to scrambled fearful avatar expressions in the second level whole-brain analysis* | | | | | | | |
| Brain area | Side | *k* | MNI coordinates | | | *T*-value | *p-FWE* |
|  |  |  | *x* | *y* | *z* |  |  |
|  |  |  |  |  |  |  |  |
| Fusiform gyrus | R | 1547 | 42 | -42 | -18 | 13.62 | < .001 |
| Inferior occipital gyrus | R |  | 38 | -78 | -6 | 12.09 | < .001 |
| Inferior occipital gyrus | R |  | 42 | -68 | -12 | 11.54 | < .001 |
| Fusiform gyrus | L | 730 | -42 | -50 | -18 | 9.5 | < .001 |
| Middle temporal gyrus,  posterior | L |  | -52 | -50 | 8 | 8.9 | < .001 |
| Fusiform gyrus | L |  | -42 | -66 | -14 | 8.24 | < .001 |
| Frontal operculum | L | 9 | -40 | 26 | 4 | 6.27 | .013 |
| Amygdala | L | 17 | -24 | -6 | -14 | 6.33 | .012 |
| *Note.* Voxel-wise FWE corrected *p*-value is shown. Missing values under *k* indicate that the activation peak of the respective brain area pertains to the above-mentioned cluster. In cases where several activation peaks belonged to one cluster, the brain areas were listed according to the size of their *T*-value. | | | | | | | |

| Supplementary Table S5 | | | | | | | |
| --- | --- | --- | --- | --- | --- | --- | --- |
| *Clusters showing a significant difference in BOLD response to neutral avatar expressions compared to scrambled neutral avatar expressions in the second level whole-brain analysis* | | | | | | | |
| Brain area | Side | *k* | MNI coordinates | | | *T*-value | *p-FWE* |
|  |  |  | *x* | *y* | *z* |  |  |
|  |  |  |  |  |  |  |  |
| Middle temporal gyrus,  posterior | R | 1079 | 54 | -60 | 8 | 10.12 | < .001 |
| Fusiform gyrus | R |  | 44 | -44 | -18 | 10.01 | < .001 |
| Middle temporal gyrus,  posterior | R |  | 52 | -62 | 0 | 9.76 | < .001 |
| Inferior occipital gyrus | L | 24 | -42 | -74 | -2 | 6.63 | .007 |
| Fusiform gyrus | L | 231 | -42 | -46 | -22 | 10.01 | < .001 |
| Fusiform gyrus | L |  | -40 | -66 | -14 | 6.35 | .012 |
| *Note.* Voxel-wise FWE corrected *p*-value is shown. Missing values under *k* indicate that the activation peak of the respective brain area pertains to the above-mentioned cluster. In cases where several activation peaks belonged to one cluster, the brain areas were listed according to the size of their *T*-value. | | | | | | | |
